# Functional Mapping of AGO-Associated Zika Virus-Derived Small Interfering RNAs in Neural Stem Cells

**DOI:** 10.3389/fcimb.2021.628887

**Published:** 2021-02-25

**Authors:** Jianxiong Zeng, Zhifei Luo, Shupeng Dong, Xiaochun Xie, Xinyan Liang, Youzhen Yan, Qiming Liang, Zhen Zhao

**Affiliations:** ^1^ Department of Physiology and Neuroscience, Keck School of Medicine, University of Southern California, Los Angeles, CA, United States; ^2^ AngelicaMadlangbayanKeck School of Medicine, Zilkha Neurogenetic Institute, University of Southern California, Los Angeles, CA, United States; ^3^ Gene Expression Laboratory, Salk Institute for Biological Studies, La Jolla, CA, United States; ^4^ Department of Immunology and Microbiology, School of Medicine, Shanghai Institute of Immunology, Shanghai Jiao Tong University, Shanghai, China; ^5^ Research Center of Translational Medicine, Shanghai Children’s Hospital, Shanghai Jiao Tong University, Shanghai, China

**Keywords:** zika virus (ZIKV), RNA interference (RNAi), viral interfering RNA (viRNA), Dicer, neural stem cells (NSCs)

## Abstract

Viral interfering RNA (viRNA) has been identified from several viral genomes *via* directly deep RNA sequencing of the virus-infected cells, including zika virus (ZIKV). Once produced by endoribonuclease Dicer, viRNAs are loaded onto the Argonaute (AGO) family proteins of the RNA-induced silencing complexes (RISCs) to pair with their RNA targets and initiate the cleavage of target genes. However, the identities of functional ZIKV viRNAs and their viral RNA targets remain largely unknown. Our recent study has shown that ZIKV capsid protein interacted with Dicer and antagonized its endoribonuclease activity, which requires its histidine residue at the 41^st^ amino acid. Accordingly, the engineered ZIKV-H41R loss-of-function (LOF) mutant virus no longer suppresses Dicer enzymatic activity nor inhibits miRNA biogenesis in NSCs. By combining AGO-associated RNA sequencing, deep sequencing analysis in ZIKV-infected human neural stem cells (NSCs), and miRanda target scanning, we defined 29 ZIKV derived viRNA profiles in NSCs, and established a complex interaction network between the viRNAs and their viral targets. More importantly, we found that viRNA production from the ZIKV mRNA is dependent on Dicer function and is a limiting factor for ZIKV virulence in NSCs. As a result, much higher levels of viRNAs generated from the ZIKV-H41R virus-infected NSCs. Therefore, our mapping of viRNAs to their RNA targets paves a way to further investigate how viRNAs play the role in anti-viral mechanisms, and perhaps other unknown biological functions.

## Introduction

Zika virus (ZIKV), a member of the *Flaviviridae* family, is a single-stranded positive-sense RNA virus. The Flavivirus genus is composed of more than 50 arthropod-borne viruses such as Dengue virus (DENV), Japanese encephalitis (JEV), West Nile virus (WNV), which all pose threats to the public health. ZIKV was originally identified in the Zika forest of Uganda in 1947 (Dick et al., 1952; Weaver et al., 2016). In 2015, ZIKV outbreaks emerged unexpectedly in the Americas, and spread swiftly to 86 countries or territories worldwide in the following years, causing more than 3 billion people subject to the risk of ZIKV transmission (Baud et al., 2017).

The most concern of ZIKV is its ability to induce fetal microcephaly and congenital Zika syndrome (Rasmussen et al., 2016; Hoen et al., 2018). For example, fetal microcephaly is linked with ZIKV infection during pregnancy (Musso et al., 2016), and thousands of infants born from ZIKV-infected mothers developed abnormally with thinner cortical layers (Orioli et al., 2017). ZIKV has an intrinsic tropism for neural stem and progenitor cells (NSCs) in cell cultures, brain organoids and fetal brain slices (Cugola et al., 2016; Dang et al., 2016; Garcez et al., 2016; Liang et al., 2016; Qian et al., 2016; Tang et al., 2016), whereas ZIKV has much lower infectivity to more differentiated immature or mature neurons (Li et al., 2016a; Muffat et al., 2018). ZIKV infection impairs NSC proliferation and differentiation, triggers cell death, and results in cerebral developmental delay (Shao et al., 2016; Shao et al., 2017; Nielsen-Saines et al., 2019; Zeng et al., 2020). Notably, mammalian multipotent stem cells, including NSCs, intrinsically produce little interferon (IFN) and response poorly to IFN treatment compare to somatic cells (Hong and Carmichael, 2013; Wu et al., 2019), and thus these cells often rely on other machineries once the virus breaches the surveillance (Ding and Voinnet, 2007).

RNA interference (RNAi) is a gene silencing mechanism at post-transcriptional level in eukaryotes and also considered as an innate antiviral immunity (Samuel et al., 2018; Guo et al., 2019). Host Dicer recognizes and cleaves the viral replicative dsRNA intermediates *via* its endoribonuclease activity to produce virus-derived small interfering RNAs (viRNAs) during RNAi processing. Subsequently, the Argonaute protein (AGO) of the RNA-induced silencing complexes (RISC) utilizes viRNAs and cleaves cognate viral RNAs in infected cells. Despite RNAi is considered as an antiviral mechanism in fungi, plants and invertebrates (Li et al., 2013; Maillard et al., 2013; Qiu et al., 2017), the physiological importance of RNAi in mammals remains elusive (Cullen and Cherry, 2013; Cullen, 2017; Berkhout, 2018; Ding et al., 2018). A recent study using deep RNA-seq showed the existence of small viral RNAs at the size of viRNAs in ZIKV-infected human progenitor cells (Xu et al., 2019), however, functional characterization of ZIKV viRNAs remain unexplored.

Our recent study has shown that ZIKV capsid specifically interacts with Dicer and inhibits its endoribonuclease activity. Consequently, ZIKV infection inhibits global miRNA production in NSCs and drives neurodevelopmental defects in mouse model. Importantly, ZIKV capsid relies on its histidine (H) at 41^st^ amino acid to antagonize the Dicer activity. Accordingly, we defined the capsid H41R mutation from histidine to arginine (R), which on longer suppresses Dicer enzymatic activity due to the loss of interaction with Dicer. As a result, the ZIKV-H41R mutant virus failed to dampen miRNA biogenesis in NSCs, nor impaired corticogenesis *in vivo* (Zeng et al., 2020). However, whether ZIKV also utilizes its capsid to dampen Dicer-dependent viRNA production remains unknown. *Via* AGO-associated RNA sequencing, deep sequencing analysis in ZIKV-infected NSCs, and target scanning by miRanda, we have now depicted AGO-associated viRNA profiles and also established a complex network between the ZIKV viRNAs and their potential viral RNA (vRNA) targets. As a result, total 29 viRNAs were mapped to the ZIKV genome, and these viRNAs are predicted to target 114 sites on ZIKV genome based on target scanning. We found that much higher levels of viRNAs were generated from this mutant ZIKV-H41R virus with reduced virulence. Therefore, our previous and current studies demonstrated that ZIKV capsid modulates Dicer-dependent miRNA and viRNA productions for viral pathogenesis.

## Materials and Methods

### Viruses and Cell Culture

ZIKV strain SPH2015 and according ZIKV-H41R mutant virus were separately rescued from the ZIKV infection clone as described previously (Zeng et al., 2020). Vero cells were cultured in DMEM with 10% FBS and 4 mM glutamine. NSCs were derived from the H9 human embryonic stem cells *via* embryoid body and rosette formation, followed by rosette selection using Neural Induction Medium from STEMCELL Technology (Catalog #05835). The NSCs were subsequently maintained as monolayer culture before infection. NSCs were plated on poly-L-Ornithine (10 µg/ml) coated plates when adherent culture was needed. ZIKV WT capsid and H41R capsid were cloned into lentivirus pCDH-cmv-mcs-ef1-puro vector as described previously (Zeng et al., 2020). Lentiviruses were generated by co-transfection of pCDH-vector, pCDH-Flag-capsid^WT^ or pCDH-Flag-capsid^H41R^ with three packaging plasmids into HEK293T cells. The culture supernatant was collected at 72 h post-transfection and purified by Lenti-X Concentrator (Clontech, #631231) and then titrated with standard colony formation assay (Zeng et al., 2020).

### siRNA Gene Silencing

Small interfering RNA (siRNA) targeting human Dicer (siRNA ID: s23754), Drosha (siRNA ID: s26490), Ago2 (siRNA ID: s25930), and negative control siRNAs were purchased from Thermo Fisher Scientific. viRNA^p18^ (AGAGUGGGACUUUGUCGUGAC) and its scramble control (GCCGUCGUGTAGTTGAGTGAA) were synthesized from Thermo Fisher Scientific. siRNAs were transfected to the human NSCs *via* Lipofectamine RNAiMAX Transfection Reagent (Thermo Fisher Scientific, #13778030) according to the manufacturer’s instructions. The knock-down efficiency was verified by quantitative RT-PCR at 48 h after transfection or replaced with fresh medium for indicated virus infection at 12 h after transfection.

### Quantification of miRNAs by Real-Time PCR

Profiling of immature or mature forms of miRNAs in NSCs was performed, as described previously (Zeng et al., 2020), using TaqMan Advanced miRNA assays (Thermo Fisher Scientific, #A25576) on StepOne Plus Real-time PCR system (Applied Biosystems) following the manufacturer’s instructions. In brief, 20 ng total RNA was used for preparing cDNA templates including poly(A) tailing, adaptor ligation, reverse transcription and miRNA amplification steps (14 cycles) using the TaqMan Advanced miRNA cDNA synthesis kit (Thermo Fisher Scientific, #A28007), following the manufacturer’s instructions. TaqMan miRNA real-time PCR reactions were performed on StepOne Plus Real-time PCR system, in triplicates for each sample. The 20 µl reaction volume consisted of 2x TaqMan Fast Advanced Master Mix (10 µl), 20x TaqMan Advanced miRNA Assay (1 µl), 4 µl nuclease free water and 5 µl of amplified miRNA. RT-PCR reactions were performed according to the standard protocol with 95°C for 20 s for enzyme activation first, and then 40 cycles of 95°C for 1 s for denature and 60°C for 20 s for annealing and extension. Assays used for mature miRNAs let-7, miR-9, and miR-17 were: hsa-let-7a-5p (assay ID: 000377), hsa-miR-9-5p (assay ID: 000583), and hsa-miR-17-3p (assay ID: 002308). Assays used for precursor miRNAs let-7, miR-9, and miR-17 were: hsa-let-7a-1 (assay ID: Hs03302533_pri), hsa-mir-9-1 (assay ID: Hs03303201_pri), and hsa-mir-17-1 (assay ID: Hs03295901_pri). Relative quantities of miRNA were determined using the Applied Biosystems real-time PCR Analysis Modules and standard ΔΔCt method, by normalizing to an endogenous control assay (U6 snRNA, assay ID: 001973).

### RNA Extraction and Quantitative RT-PCR

Total RNA prepared from cells using the RNeasy Mini Kit (Qiagen) was treated with RNase-free DNase according to the manufacturer’s protocol. Complementary DNA was reversely transcribed from 1 μg of the prepared RNA using the ProtoScript First Strand cDNA Synthesis Kit (NEB) and qPCR was conducted with iQ SYBR Green Supermix (Bio-Rad). Primer sequences for qPCR were as follows: human GAPDH, sense: GAGTCAACGGATTTGGTCGT, anti-sense: TTGATTTTGGAGGGATCTCG; human Dicer, sense: TTAACCTTTTGGTGTTTGATGAGTGT, anti-sense: GCGAGGACATGATGGACAATT; human Drosha, sense: CATGTCACAGAATGTCGTTCCA, anti-sense: GGGTGAAGCAGCCTCAGATTT; human Ago2, sense: CCGGCCTTCTCTCTGGAAAA, anti-sense: GCCTTGTAAAACGCTGTTGCT.

### AGO-Associated RNA Preparation

AGO HITS-CLIP was previously utilized to map AGO-bound miRNA (Chi et al., 2009; Clark et al., 2014). To discriminate functional ZIKV-derived viRNAs from ZIKV genome, we conducted the crosslinking and immunoprecipitation (CLIP) with minor modifications. Briefly, NSCs were grown to 90% confluency and infected ZIKV at a MOI of 1 for 48 h. At 24 hpi, Protein A Dynabeads (Invitrogen) were washed three times with PBS and resuspended in antibody binding (AB) buffer (1×PBS, 0.02% Tween-20). Per cell sample, 150 μl of the beads was used. Beads were then incubated with 8 μl of pan-Ago antibody (2A8, EMD Millipore) or rabbit anti-mouse IgG antibody (Cat#315-005-008 from Jackson ImmunoResearch) as an isotype non-specific control. Beads were then rotated for overnight at 4°C. After 48 h post ZIKV infection, NSCs were washed twice with PBS and UV irradiated at 254 nm for a total energy dispersion of 600 mJ/cm^2^ in a Stratalinker XL-500 (Stratagene). Cells were lysed with 1×PXL (1×PBS, 0.1% SDS, 0.5% deoxycholate, 0.5% NP-40, protease inhibitors). Cell lysates were treated sequentially with RNAsin (Promega) and RQ1 DNase (Promega) prior to co-immunoprecipitation of RNA-protein complexes on the protein A Dynabeads for 4 h at 4°C. Beads were then washed twice each with 1×PXL, 5×PXL (5×PBS, 0.1% SDS, 0.5% deoxycholate, 0.5% NP-40, protease inhibitors), and 1×PNK buffer (50 mM Tris-Cl, 10 mM MgCl_2_, 0.5% NP-40, protease inhibitors). CLIP-RNAs were liberated with their on-bead protein complexes by treatment with 4 mg/ml protease K (Sigma) and subsequent phenol/chloroform extraction as described previously (Chi et al., 2009). miRNA libraries of CLIP-RNA were constructed and sequenced on HiSeq3000. As the CLIP-RNAs in the IgG antibody group were undetectable using Nanodrop 2000 Spectrophotometers or when analyzed by the Agilent small RNA bioanalyzer, the sample was not included in subsequent library construction and sequencing.

### AGO-Associated RNA Sequencing

Primary sequencing data were examined by FastQC version 0.11.7 for quality control purpose. Reads were trimmed by cutadapt version 1.16 (Martin, 2011) to remove adapter sequences with at least 5bp overlap (-a AACTGTAGGCAC -O 5 -m 15 –max-n 0). To increase accuracy, reads have N base or are shorter than 15bp, the minimal length of miRNA in miRbase, were removed. Trimmed reads were first mapped to human genome GRCh38 and the reads do not match human genome were mapped against Zika virus genome (KU321639.1) using Bowtie version 1.2.2 (Langmead et al., 2009) with option -n 0 -l 18 -a –best –strata to find the best aligned reads. Seed length for alignment was set as 18 and no mismatch was allowed in seed region. Peak calling to define the viRNA loci was carried out by HOMER package (Heinz et al., 2010) as previous researches (Luna et al., 2017). Explicitly with the following setting: findPeaks -style factor -size 27 -fragLength 22 -tagThreshold 5 -minDist 20 -o auto -LP 0.05 -L 2. Reads on positive strand and negative strand were used to define positive strand or negative strand viRNA separately. viRNAs loci with overlapped coordinates were manually split by visualizing read distribution in integrative genomics viewer. The common viRNA peaks among different samples was determined by mergePeaks. More specifically, any reads with middle position didn’t fall in the peak regions were discard for downstream comparison analysis. The Zika virus secondary structure was predicted by RNAfold with minimum free energy (MFE) and partition function (Lorenz et al., 2011). The figure was colored by base-pairing probability and visualized with virus miRNA highlighted using forna tool (http://rna.tbi.univie.ac.at/forna/).

### Small RNA Library Construction

As described previously (Zeng et al., 2020), 1x10^6^ NSCs that were infected with ZIKV at MOI of 1 were subject to total RNA extraction at 2 dpi. The construction of miRNA sequencing library was performed by QIAseq miRNA Library Kit (Qiagen, #331505) by UCLA Technology Center for Genomics & Bioinformatics (TCGB), followed by deep sequenced using Illumina HiSeq3000. Briefly, the workflow consists of 3’ ligation, primer hybridization, 5’ ligation, first strand synthesis and PCR amplification. Different adapters were used for multiplexing samples in one lane. AMPure XP Beads were used for dual size selection. Library quality was checked with Bioanalyzer (Agilent) and Qubit (Life Technologies).

### Small RNA Sequencing

As described previously (Zeng et al., 2020), primary sequences data were processed as AGO HITS-CLIP data. Specifically, it was checked by FastQC version 0.11.7 for quality control purpose. Reads have N base were removed by cutadapt version 1.16 (Martin, 2011) (–max-n 0). Trimmed reads were mapped to human genome GRCh38 using Bowtie version 1.2.2 (Langmead et al., 2009) with option -n 0 -l 17 -a –best –strata to find the best aligned reads. Seed length for alignment was set as 17 and no mismatch was allowed in seed region. Reads that failed to align to human genome were mapped to ZIKV genome. The reads were assigned to defined viRNA if the middle position of the read is within the viRNA.

### Quantification of viRNA by Real-Time PCR

Quantification of viRNA was conducted by Taqman assay as described previously (Zeng et al., 2020) with minor modification. Profiling of viRNA in ZIKV infected human NSCs was performed using TaqMan Advanced miRNA assays (Thermo Fisher Scientific, #A25576) on StepOne Plus Real-time PCR system (Applied Biosystems) following the manufacturer’s instructions. In brief, 5x10^5^ NSCs or Drosha- or Dicer-knockdowned NSCs were infected with ZIKV-WT or ZIKV-H41R at a MOI of 1, and small RNA was extracted by mirVana miRNA kit at 48 hpi for quantification of viRNA copy.

1 ng of small RNA was used for preparing cDNA templates including poly(A) tailing, adaptor ligation, reverse transcription and miRNA amplification steps (14 cycles) using the TaqMan Advanced miRNA cDNA synthesis kit (Thermo Fisher Scientific, #A28007), following the manufacturer’s instructions. TaqMan miRNA real-time PCR reactions were performed on StepOne Plus Real-time PCR system, in triplicates for each sample and each miRNA. The 20 µl reaction volume consisted of 2x TaqMan Fast Advanced Master Mix (10 µl), 20x TaqMan Advanced miRNA Assay (1 µl, customed by Thermo Fisher Scientific), 4 µl nuclease free water and 5 µl of amplified miRNA with 10-fold dilution. RT-PCR reactions were performed according to the standard protocol with 95°C for 20 s for enzyme activation first, and then 40 cycles of 95°C for 1 s for denature and 60°C for 20 s for annealing and extension. For viRNA Taqman positive control, 21 nt-long single-strand RNA corresponding to viRNA^p18^ (5-AGAGUGGGACUUUGUCGUGAC-3’) was synthesized with termination of 5’PO_4_ and OH-3’ in the condition of RNase free HPLC by Integrated DNA Technologies. Similarly, 1 ng (theoretically containing 9.0 x 10^10^ copies of 21nt-long ssRNA copies) of synthesized ssRNA was used for miRNA cDNA synthesis and Taqman miRNA real-time PCR. Accordingly, copy of the viRNA^p18^ in total small RNAs purified from 5 x 10^5^ NSCs can be calculated, and the resultant total copies of the viRNA^p18^ divided by cell numbers (5 x 10^5^) produce the viRNA^p18^ copy per cell. Taqman probes and assays are not intercalating dyes and melt curves do not need to be performed. The probe is designed to anneal specific to target of interest which minimizes primer dimers and/or nonspecific PCR product detection.

### viRNA Target Prediction by miRanda

The viRNA target scanning was performed by miRanda v3.3a with default setting. Sequences of viRNAs predicted on either positive or negative strand were used to for target prediction against either ZIKV genome or its reverse complementary sequences.

### Viral Plaque Assays

Viral plaque assays were performed as described previously (Zeng et al., 2020). In briefly, cells were plated at a density of 5.0 × 10^4^ cells/well in 1 ml DMEM, 10% FBS, on 24-well plates and incubated at 37°C, 5% CO_2_ atmosphere. On the next day, 300 μl of appropriate ten-fold dilutions (usually ranging from undiluted to 10^-7^-fold diluted) of supernatants from infected cultures were added to each well. After incubation of 1.5 h, supernatants were discarded and the Vero cells layer were rinsed and overlaid with 0.7% methylcellulose. After incubation of 4 days, the overlaid methylcellulose was discarded, and the Vero cell layer was fixed and stained with crystal violet. Virus content of the supernatants was calculated as plaque forming units (PFU)/ml.

### Statistical Analysis

All data were expressed as Mean ± SD. For parametric analysis, the F test was used to determine the equality of variances between the groups compared; statistical significance across two groups was tested by Student’s *t*-test; one-way analysis of variance (ANOVA) followed by Bonferroni’s *post hoc* test were used to determine statistically significant differences between multiple groups. *P*-values of less than 0.05 were considered significant.

## Results

### AGO-Associated viRNA Profiles in ZIKV-Infected NSCs

**Figure 1:**
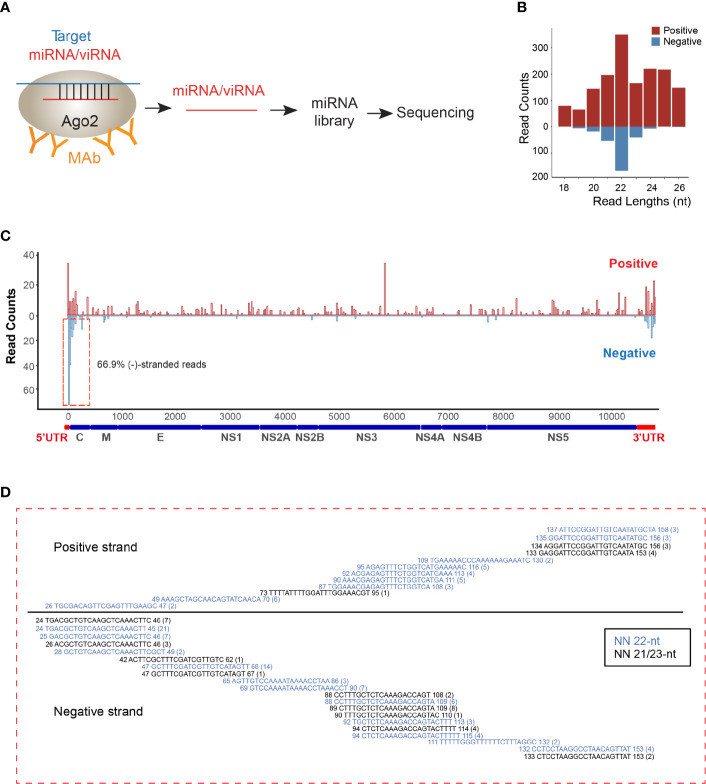
viRNAs are identified *via* AGO-associated RNA sequencing in ZIKV-WT-infected NSCs. **(A)** Schematic diagram for AGO-associated RNA sequencing. **(B)** Size distribution of viRNAs in AGO-associated RNA sequencing in ZIKV-WT-infected NSCs (MOI:1, 48 h post-infection). Red, positive-stranded viRNAs; blue, negative-stranded viRNAs. **(C)** The distribution and relative abundances of viRNA reads (21~23 nt) from AGO-associated RNA sequencing on the ZIKV genome (Red) and complementary sequence (blue). **(D)** Read sequence along 5’UTR region of ZIKV genome. Read counts (in brackets), read length, and genomic position are indicated.

As viRNAs are loaded into the RISC for pairing with their RNA targets and then initiating cleavage of the target genes, we decided to perform AGO-associated RNA sequencing in ZIKV-infected NSCs (**Figure 1A**). We analyzed overall small RNAs (< 50 nt) that are pulled out by endogenous AGO proteins with specific antibody (Clone 2A8) (Chi et al., 2009). Consistent with previous AGO-CLIP data (Chi et al., 2009), 53% of AGO-associated miRNAs can be mapped onto 3’UTR of human mRNA transcripts (**Supplementary Figures 1A, B** **)**. The read lengths of viRNAs identified were enriched to 22 ± 1 nt (**Figure 1B**), which is the size of human Dicer products (Chi et al., 2009). Mapping of the 22 ± 1 nt viRNA reads showed that they were unequally originated from positive (73%) and negative strands (27%) (**Figure 1C**). The majority of these negative-stranded viRNAs reads were generated from the terminal regions of antigenomic RNAs, and these viRNAs at the terminal formed continuous complementary pairs with their counter partners mapped on positive strand (**Figure 1D**), suggesting that these viRNAs were generated by Dicer from viral dsRNA intermediates as previously reported (Li et al., 2016b; Qiu et al., 2017). As a result, total 29 viRNAs were identified across ZIKV genome (**Supplementary Table 1**). 88.04% of the identified viRNAs were mapped to the coding region of the positive strand in ZIKV genome (**Figure 1C**).

### The Mapping of viRNAs and Their Viral Targets by the miRanda Scanning

**Figure 2:**
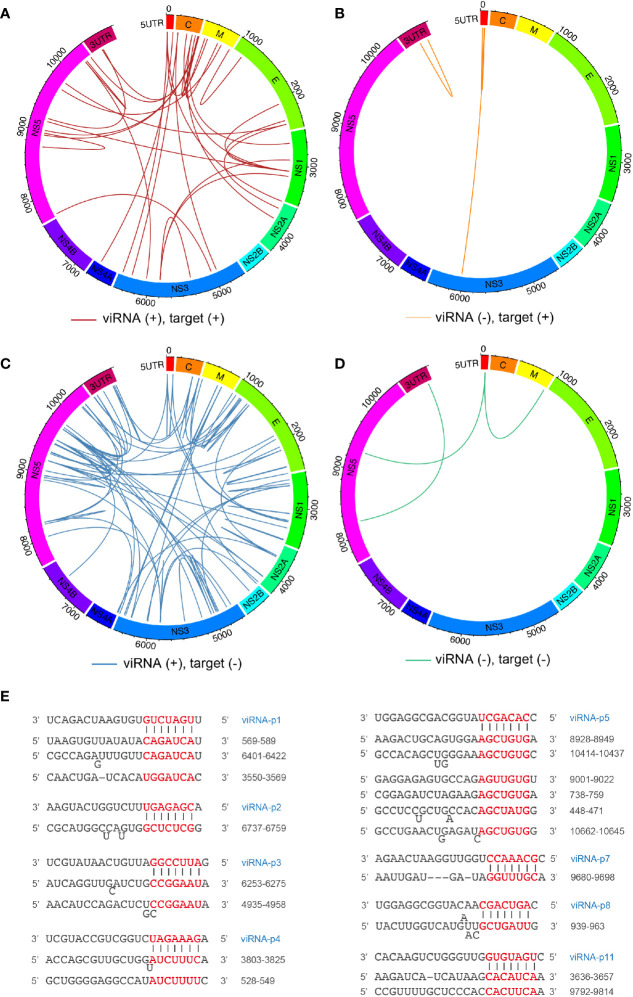
Mapping ZIKV viRNAs-viral RNA interactome *via* miRanda target scanning. **(A–D)** Circos plot of long-range interaction between viRNA from ZIKV positive (+)- or negative (-)-strand and its predicted viral targets from viral positive (+)- or negative (-)-strand by miRanda (omictools.com/miRanda-tool) in ZIKV (SPH2015) genome. In details, **(A)** (31 pairings): viRNA(+)-target (+); **(B)** (four pairings): viRNA(-)-target(+); **(C)** (76 pairings): viRNA(+)-target(-); **(D)** (three pairings): viRNA(-)-target(-); **(E)** Paring between representative viRNAs identified and its viral targets predicted by miRanda.

Next, we mapped the 29 viRNAs with their potential ZIKV RNA targets. The targeting efficiency of viRNA is defined not only by nucleotide sequence property but also by the surrounding secondary structure of their targeting sequences. Therefore, we systematically predicted the viRNA-viral target interaction using the miRanda software (Betel et al., 2010). Overall, we identified 114 viRNA-viral target interactions (**Supplementary Table 2**), and found 30.7% and 69.2% viRNA targets are located on the positive (**Figures 2A, B** **)** and negative (**Figures 2C, D** **)** strands, respectively. Similar to host miRNAs, viRNAs utilize a seed sequence (nucleotides 2-8 from 5’ of viRNA) to recognize complementary target mRNAs (Bartel, 2009), as shown by several representative viRNA-vRNA target pairings (**Figure 2E**). These analytic results indicate that a complexed viRNA-dependent host-viral inhibition network exist in NSCs during ZIKV infection.

### viRNA Production Is Dicer-Dependent

**Figure 3:**
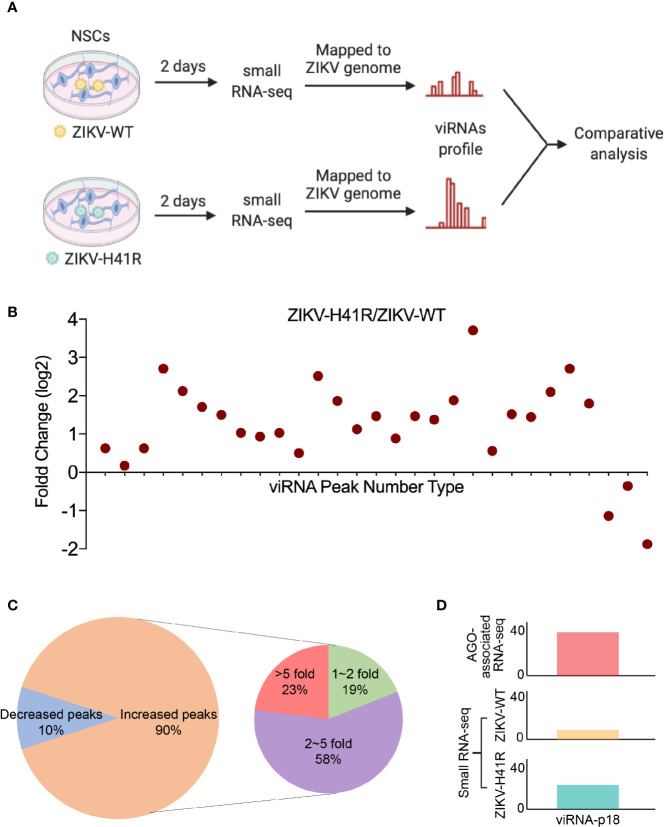
The identified viRNAs are potentially the products of Dicer activity *via* directly deep small RNA-seq of ZIKV-infected NSCs. **(A)** Schematic diagram of comparative analysis for directly deep small RNA-seq in ZIKV-WT- or ZIKV-H41R-infected NSCs. **(B)** Comparison of read counts in 29 ZIKV viRNA peaks in ZIKV-WT- or ZIKV-H41R-infected NSCs at MOI of 1 for 2 days. The fold change of presented peaks was log2 scale. **(C)** 90% viRNA peaks identified from AGO-associated RNA sequencing are increased in ZIKV-H41R infected NSCs compared to ZIKV-WT infected ones. **(D)** Peak landscape of representative viRNA-p18 in AGO-associated RNA-seq and deep small RNA-seq. Value in the small RNA-seq (lower panel) in y-axis indicates reads per 10 million sequenced reads.

Since Dicer has been considered to be critical for ZIKV viRNA production (Xu et al., 2019), and we previously defined the H41R LOF mutation that no longer inhibits Dicer, we next performed direct deep RNA sequencing of NSCs infected with ZIKV-WT- or ZIKV-H41R virus. *Via* analyzing small RNA profiles (**Figure 3A**), we found that the 29 viRNA peaks identified in the AGO-associated RNA sequencing are all present (**Figure 3B**). More importantly, much higher levels of viRNAs were generated from NSCs infected with the ZIKV-H41R virus (**Figure 3B**). The detailed analysis indicated that ~90% of the viRNA profiles were upregulated in ZIKV-H41R infection when compared with ZIKV-WT infection (**Figure 3C**, **Supplementary Table 3**). As an example, viRNA-p18 (viRNA^p18^), the highest profile based on the AGO-associated RNA-seq (**Figure 1C**), was produced 2.59-fold higher in the ZIKV-H41R virus-infected NSCs (**Figure 3D**). These results suggest that the viRNAs identified in AGO-associated RNA sequencing were products of Dicer, and capsid-mediated inhibition of Dicer and viRNA production is key to its virulence.

### viRNA Is a Limiting Factor for ZIKV Infection in NSCs

**Figure 4:**
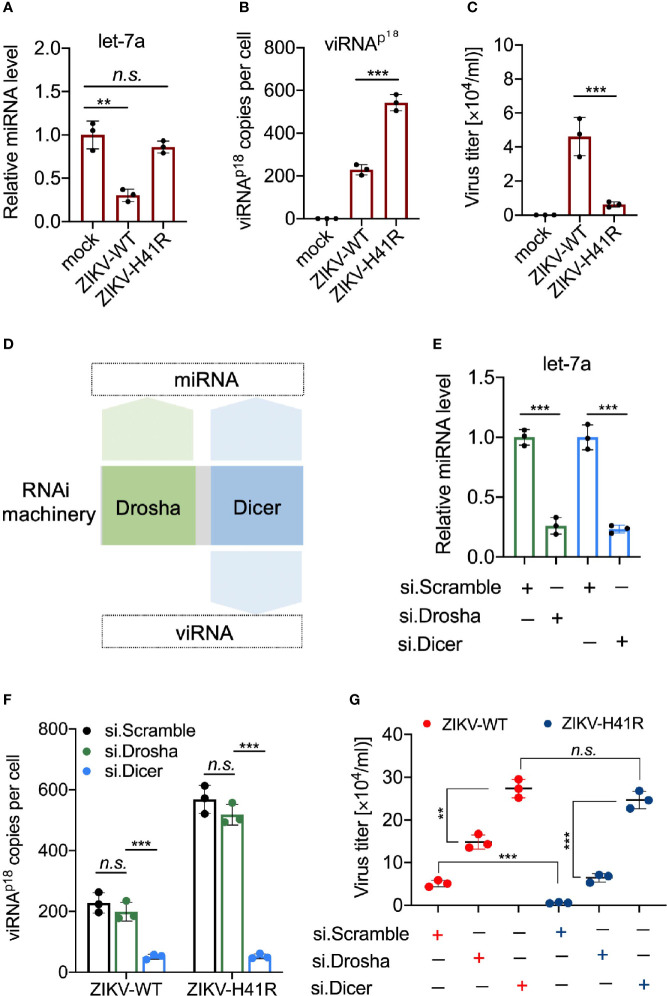
Dicer-dependent viRNAs are potentially anti-viral in ZIKV-infected NSCs. **(A)** Taqman Advanced miRNA assays for miRNA let-7a in NSCs infected with ZIKV-WT or ZIKV-H41R. Mean ± SD; **p < 0.01 by one-way ANOVA and Bonferroni’s *post hoc* test. n.s., not significant. **(B)** Customed Taqman Advanced assays for viRNA-p18 (viRNA^p18^) detection in NSCs infected with ZIKV-WT or ZIKV-H41R. Mean ± SD; ***p < 0.001 by one-way ANOVA and Bonferroni’s *post hoc* test. **(C)** NSCs were infection with ZIKV-WT or ZIKV-H41R mutant virus at MOI of 0.01, and culture supernatant were collected for virus titer determined by plaque assay. **(D)** Schematic diagram for the involvement of RNAi machinery components in miRNA or viRNA biogenesis. **(E)** Taqman Advanced miRNA assays for mature miRNA let-7a in scramble, Drosha, or Dicer siRNA-treated NSCs. Mean ± SD; ***p < 0.001 by Student’s *t*-test. **(F)** viRNA generation is dependent on Dicer function. 5x10^5^ NSCs transfected with si.scramble, si.Drosha, or si.Dicer for 24 h were infected with ZIKV-WT or ZIKV-H41R at MOI of 0.1, and representative viRNA^p18^ expression was detected by customed TaqMan Advanced assays (see *Materials and Methods*). Mean ± SD; ***p < 0.001 by one-way ANOVA and Bonferroni’s *post hoc* test. n.s., not significant. **(G)** Both miRNA and viRNA play important role to limit ZIKV replication in NSCs. 5 x10^5^ NSCs were individually transfected with si.scramble, si.Dicer, or si.Drosha for 24 h, followed by infection with ZIKV-WT or ZIKV-H41R mutant virus at MOI of 0.01, and culture supernatant were collected for virus titer determined by plaque assay. Mean ± SD; **p < 0.01 and ***p < 0.001 by Student’s *t*-test. n.s., not significant.

We recently reported that ZIKV infection suppresses the host RNAi machinery in NSCs (Zeng et al., 2020). For example, ZIKV-WT infection significantly reduced mature miRNA production in NSCs, including let-7a (**Figure 4A**), miR-9 (**Supplementary Figure 2A**), and miR-17 (**Supplementary Figure 2B**), compared to ZIKV-H41R infection. For quantitative detection of individual viRNA in ZIKV-infected NSCs, we designed and customized Taqman assays, which allow us to calculate viRNA copies per cell (see Method). As a result, we found that the representative viRNA^p18^ was detectable in ZIKV-WT infected NSCs, and its level was 2.37-fold higher in ZIKV-H41R infected NSCs (**Figure 4B**). Consequently, ZIKV-H41R mutant virus has much lower replication capacity than ZIKV-WT in NSCs (**Figure 4C**).

Next, we determined the anti-viral effects of miRNAs and viRNAs in NSCs. As Drosha is only responsible for precursor miRNA (pre-miRNA) generation in nucleus upstream of Dicer, it only participates in miRNA production, while Dicer and Ago2 govern both miRNA and viRNA functions (**Figure 4D**). Thus, we hypothesize that combining ZIKV-H41R mutant with individually knockdown of Drosha or Dicer will allow us to tease out the anti-ZIKV roles of miRNA and viRNA in NSCs. As expected, Drosha knockdown dampened pre-miRNAs including pre-let-7a, pre-miR-9 and pre-miR-17 (**Supplementary Figures 3A, B** **)**, while knockdown of Dicer significantly impaired mature miRNA production including let-7a (**Figure 4E**) and miR-9/miR-17 (**Supplementary Figure 3C**). In addition, Drosha knockdown has no effect on viRNA^p18^ production in either ZIKV-WT or ZIKV-H41R infected NSCs (**Figure 4F**), indicating that Drosha is not involved in viRNA biogenesis. More importantly, knockdown of Dicer not only significantly reduced viRNA^p18^ production but also normalized the difference of viRNA production between ZIKV-WT and ZIKV-H41R (**Figure 4F**). On the other hand, Drosha knockdown only led to increased viral burdens of both ZIKV-WT and ZIKV-H41R viruses, but failed to normalize the difference between both (**Figure 4G**), suggesting that both miRNA and viRNA systems have antiviral effects in NSCs upon ZIKV infection.

To further reveal the RNAi suppression activity of ZIKV capsid, we examined the viRNA^p18^ level in ZIKV-infected NSCs with ectopically expression of capsid^WT^ or capsid^H41R^. The ectopic expression of capsid^WT^ but not capsid^H41R^ reduced the viRNA^p18^ production in ZIKV-WT- or ZIKV-H41R-infected NSCs (**Supplementary Figure 4**). To preliminarily determine the physiological importance of the viRNA^p18^, we transfected NSCs with synthesized viRNA^p18^ mimetic RNA (viRNA^p18^) and found that it reduced ZIKV burden significantly, when compared to a scramble control (vi.scramble) (**Supplementary Figure 5**), suggesting the antiviral potential of individual viRNA in NSCs.

In sum, our data demonstrated that both miRNA and viRNA systems are part of the antiviral response during ZIKV infection in NSCs, and targeting these pathways may offer new therapeutic approaches.

## Discussion

RNAi is a main antiviral mechanism in fungi, plants and invertebrates. Due to the undetectable viRNAs in mammalian somatic cells infected with some wild-type human viruses such as EV71, IAV, and Sindbis virus (Parameswaran et al., 2010; Girardi et al., 2013; Backes et al., 2014; Bogerd et al., 2014), the role of RNAi as a critical mammalian antiviral defense mechanism remains under-debate. The recent study *via* direct deep RNA-seq has shown that ZIKV infection generated detectable production of viRNAs in human NPCs, which is comparable to mosquito Aag2 cells (Xu et al., 2019). In the present study, we combined AGO-associated RNA-seq with direct deep RNA-seq and identified 29 potentially functional viRNAs in ZIKV-infected NSCs. The utilization of the mutant H41R highlighted that the viRNA production across ZIKV genome is associated with Dicer function. Our results also demonstrated that that ZIKV capsid is a viral suppressor of RNAi (VSR) *via* targeting Dicer directly. Therefore, the identification of the AGO-associated viRNAs in the present study paves a way to investigate physiological antiviral function of individual viRNA in mammals. In addition, it is reasonably to speculate that viRNAs achieve their biological function by targeting host genes. Considering the potential complexity of viRNA-host targets, we first studied how viRNAs target viral genome to achieve their potential antiviral function. Comprehensive analysis and functional exploration for viRNAs-host targets are scheduled in future.

The universal detectable viRNAs in fungi, plants, and invertebrates makes a logical hypothesis that viRNAs may be also readily detectable and physiologically important in mammals. However, the successive studies on a group of wild-type human viruses including IAV, EV71, and Sindbis virus have failed to detect viRNAs in mammalian somatic cells. Such negative results were suspected to be caused by virus encoded VSRs, as the VSR-defective viruses in fact can produce detectable viRNAs in mammalian somatic cells (Ding et al., 2018). The potential interaction between interferon response and RNAi pathway is likely to be another reason, as in somatic cells interferon represents a main anti-viral immunity to antagonize pathogenic agent. ZIKV replicates preferentially in NSCs with poor interferon response, which provides a unique opportunity to detect viRNAs and characterize their biological functions.

Mammalian multipotent stem cells largely rely on RNAi machineries as antiviral surveillance mechanism (Ding and Voinnet, 2007). Interestingly, histidine at the 41^st^ position is crucial for ZIKV capsid to suppress RNAi machineries including miRNA and viRNA, which benefits ZIKV pathogenesis in progenitor cells of fetal brain. Nevertheless, other flaviviruses primarily target somatic cells and have evolutionarily developed complicated strategies to counter IFN pathway. In addition, flaviviruses have also evolved different ways to antagonize the Dicer-mediated viRNA mechanism. For example, viral proteins 3A, NS1, NS2A, and capsid have been identified as the VSRs for human EV71, IAV, DENV, and SFV, respectively (Qiu et al., 2017; Tsai et al., 2018; Qian et al., 2020; Qiu et al., 2020). Accordingly, the VSR-defective mutant viruses can produce abundant viRNAs in mammalian cells. Our recent study identified that ZIKV capsid targets Dicer and suppresses its ribonuclease activity, while the Dicer-binding defective H41R mutant lost this function (Zeng et al., 2020). As a result, much more abundant viRNA productions across ZIKV genome were observed in ZIKV-H41R-infected NSCs, suggesting that the capsid is a new VSR of ZIKV.

Recent study have demonstrated that the well-known RNAi enhancer, enoxacin, substantially ameliorated ZIKV-induced microcephaly in brain organoids (Xu et al., 2019), suggesting that combating ZIKV invasion largely depends on RNAi immunity at early stage of human brain development. Nevertheless, the physiological role of RNAi in mammals remains elusive. There was a prevailing view that viRNAs are produced by Dicer cleavage of viral replicative dsRNA intermediates, as the viRNA reads often fall in the untranslated regions (UTR) based on direct sequencing (Xu et al., 2019). However, the peaks in the middle of viral genome are often strand-specific, yet remain unexplored. Because individual RNAi such as viRNA and miRNA exerts their function only when loaded into the AGO, AGO-associated RNA-seq is likely to identify true functional viRNAs (Ding et al., 2018). Our results have shown that most of viRNA peaks were mapped to the positive strand and in the middle of ZIKV genome, suggesting they are likely produced through a different mechanism, which should be determined in future studies.

## Data Availability Statement

The datasets generated during this study were deposited to Gene Expression Omnibus (GEO) repository with the accession number GEO: GSE159916.

## Author Contributions

ZZ, QL, and JZ conceived the research and designed the study. ZZ and JZ wrote the manuscript. ZL analyzed the sequencing data. JZ, SD, XX, XL, and YY performed the experiments. All authors contributed to the article and approved the submitted version.

## Funding

This work was supported by NIH grant R01NS110687 to ZZ.

## Conflict of Interest

The authors declare that the research was conducted in the absence of any commercial or financial relationships that could be construed as a potential conflict of interest.
