# Supplementary material for: Functional Mapping of AGO-Associated Zika Virus-Derived Small Interfering RNAs in Neural Stem Cells
Source: Front Cell Infect Microbiol. 2021 Feb 25;11:628887. doi: 10.3389/fcimb.2021.628887 (PMC7946837; doi:10.3389/fcimb.2021.628887)
Supplement: Supplementary file 1 [file DataSheet_1.docx]

**Supplementary figures**

**Functional mapping of AGO-associated zika virus-derived small interfering RNAs in neural stem cells**

Jianxiong Zeng^1,2,6^, Zhifei Luo^3,6^, Shupeng Dong^4,5^, Xiaochun Xie^1,2^, Xinyan Liang^1,2^, Youzhen Yan^1,2^, Qiming Liang^4,5^, and Zhen Zhao^1,2,7^*


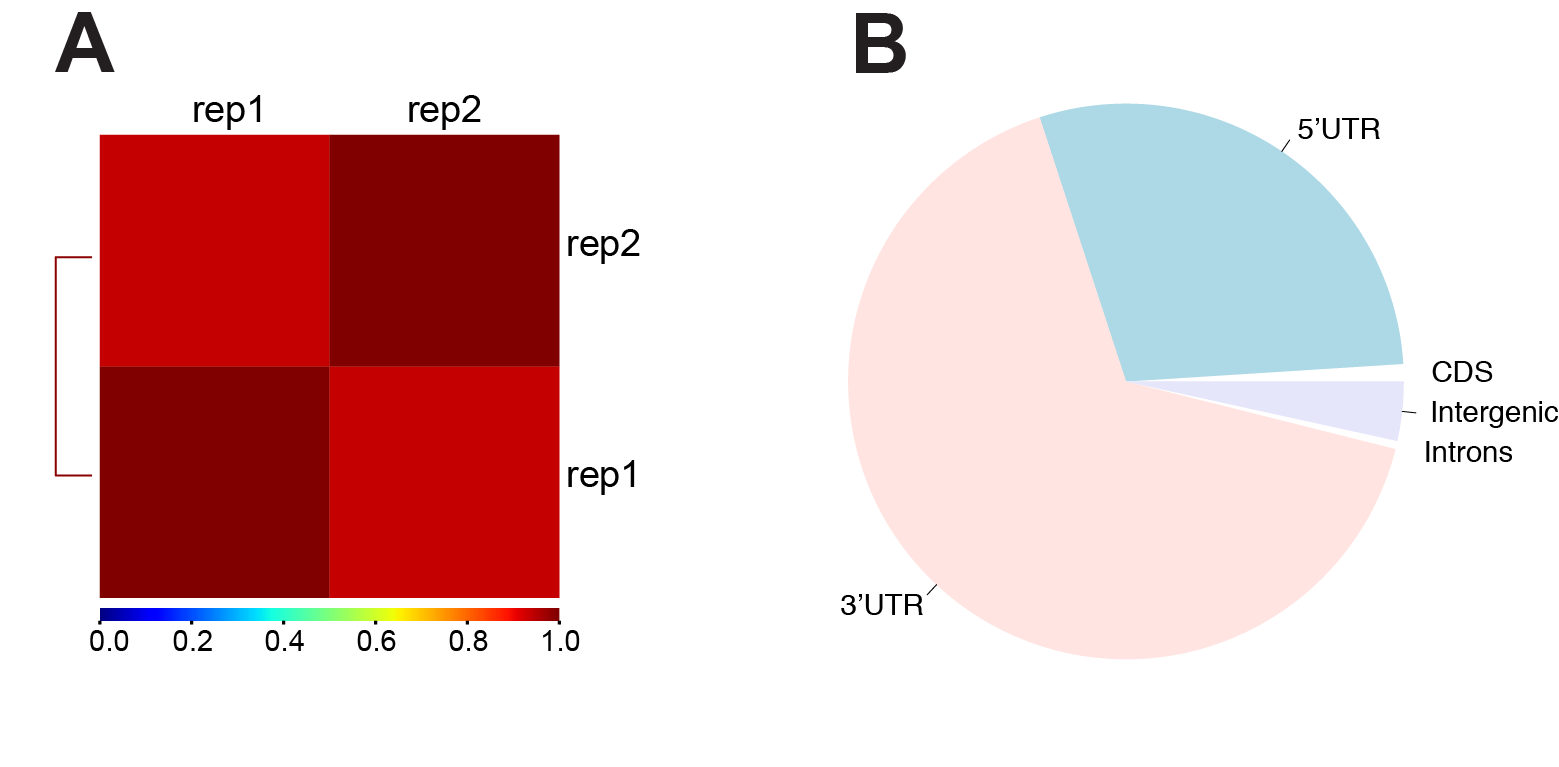


**Supplementary Figure 1.** **Other characteristics of AGO-associated RNA sequencing**. (**A**) Shown is correlation analysis (pearson score 0.94) of reads mapped to human genome from two independent replicates of AGO-associated RNA sequencing. (**B**) Location of AGO-associated RNA sequencing reads in the genome. Reads were mapped to human genome and location summarized by read_distribution.py python scripts from RSeQC packages. Average percent from two replicates were plotted.


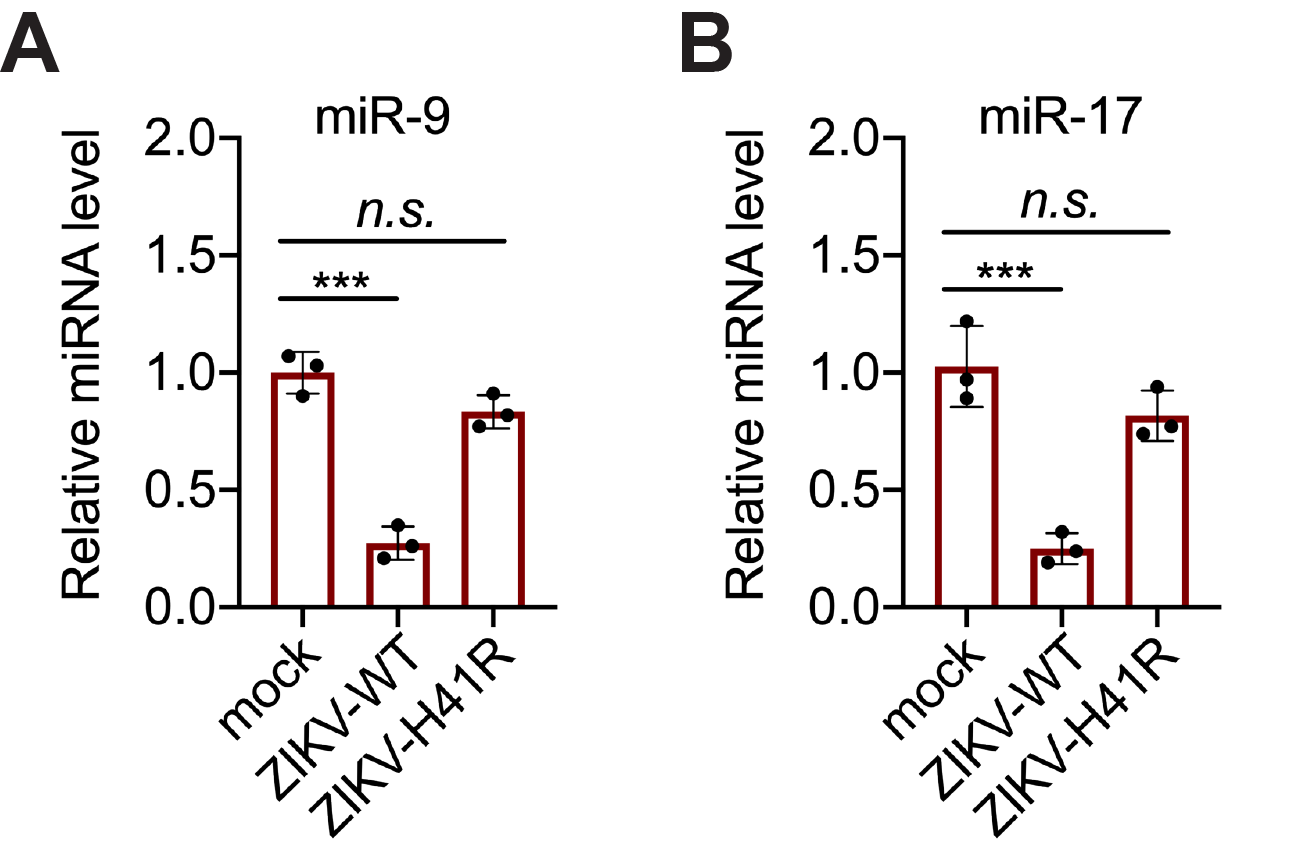


**Supplementary Figure 2.** **ZIKV-WT but no ZIKV-H41R inhibited mature miRNA production in NSCs.** (**A-B**) Taqman Advanced miRNA assays for miRNA miR-9 (A) or miR-17 (B) in NSCs infected with ZIKV-WT or ZIKV-H41R. Mean ± SD; ****p* < 0.001 by one-way ANOVA and Bonferroni’s *post* *hoc* test.

**
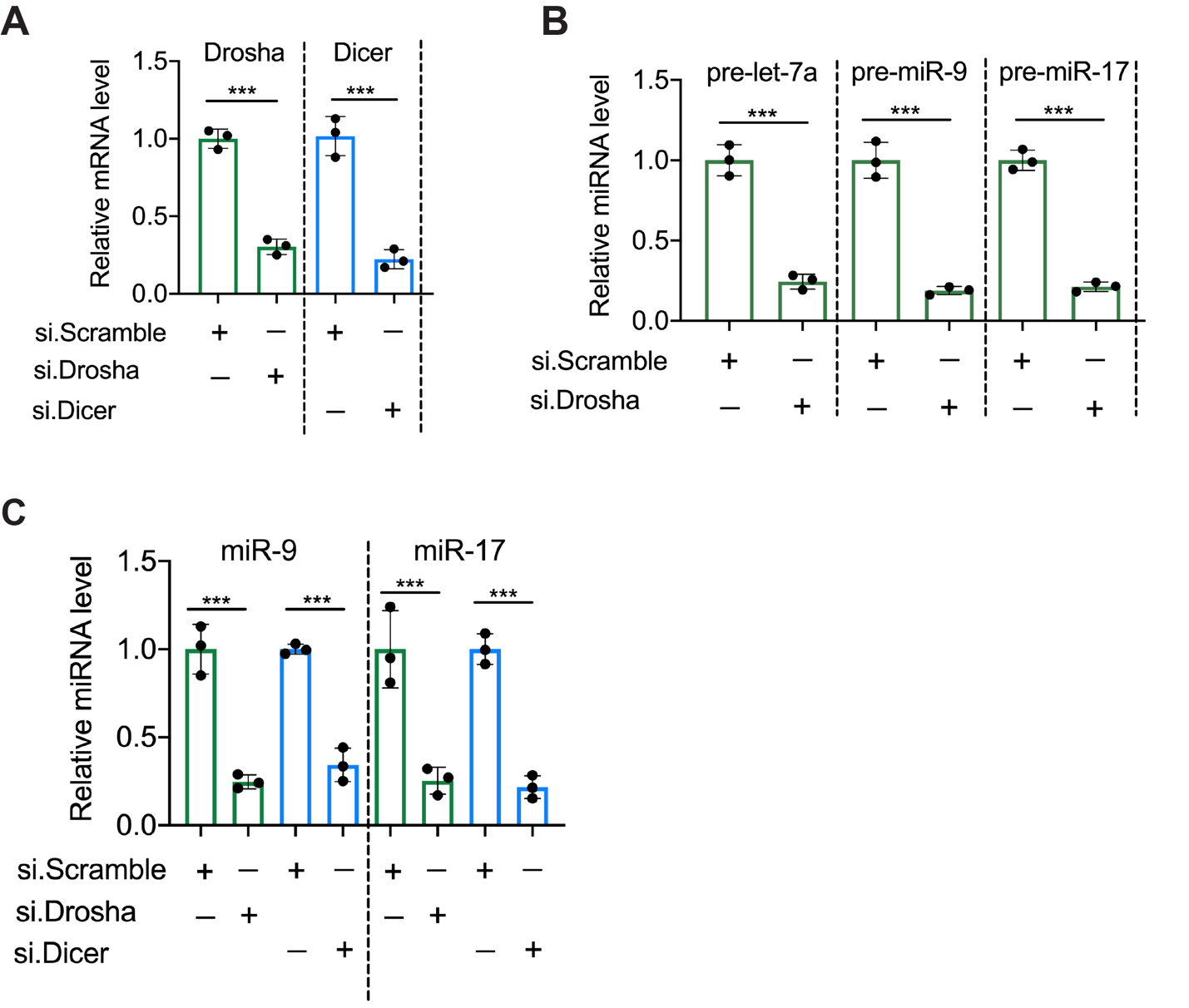
**

**Supplementary Figure 3.** **Drosha and Dicer are necessary for pre-miRNA and mature miRNA production in NSCs, respectively.** (**A**) RT-qPCR for mRNA of individual Dicer or Drosha in NSCs transfected with siRNA specifically targeting Dicer or Drosha. Mean ± SD; ****p* < 0.001 by Student’s *t*-test. (**B**) Taqman Advanced miRNA assays for pre-miRNAs including pre-let-7a, pre-miR-9, and pre-miR-17 in Drosha siRNA-treated NSCs. Mean ± SD; ****p* < 0.001 by Student’s *t*-test. (**C**) Taqman Advanced miRNA assays for mature miRNAs miR-9 and miR-17 in scramble, Drosha, or Dicer siRNA-treated NSCs. Mean ± SD; ****p* < 0.001 by Student’s *t*-test.


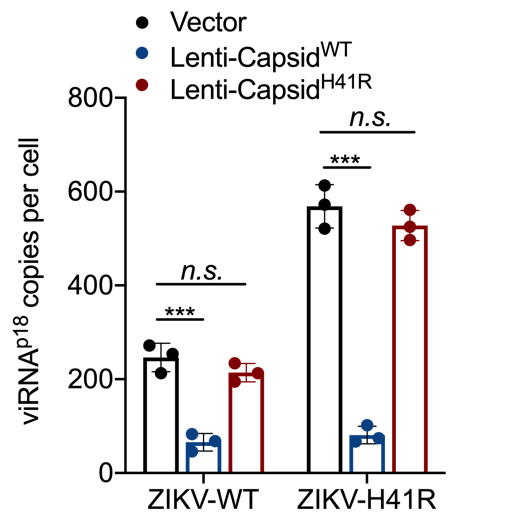


**Supplementary Figure 4. Lentivirus-mediated ectopically expression of ZIKV capsid reduced viRNA^p18^ level in NSCs.** NSCs stably expressing vector, Flag-capsid^WT^, or Flag-capsid^H41R^ were infected with ZIKV-WT or ZIKV-H41R (MOI: 0.1), and the viRNA^p18^ expression was detected by customed TaqMan Advanced assays (See Methods). Mean ± SD; ****p* < 0.001 by one-way ANOVA and Bonferroni’s *post* *hoc* test.


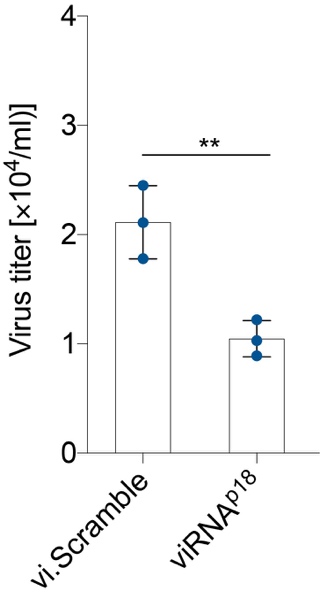


**Supplementary Figure 5. viRNA^p18^ has anti-ZIKV activity in NSCs.** NSCs were transfected with scramble viRNA (vi.scramble) and viRNA^p18^ using Lipofectamine RNAiMAX Transfection Reagent. The transfected NSCs were infected with ZIKV at MOI of 0.01, and viral titer was detected by plaque assays. Mean ± SD; ***p* < 0.01 by Student’s *t*-test.
